# Exam-related unwanted intrusive thoughts and related neutralizing behaviors: Analogues to obsessions and compulsions

**DOI:** 10.1371/journal.pone.0270692

**Published:** 2022-07-05

**Authors:** Martin Kollárik, Carlotta V. Heinzel, Marcel Miché, Roselind Lieb, Karina Wahl

**Affiliations:** Department of Psychology, Division of Clinical Psychology and Epidemiology, University of Basel, Basel, Switzerland; Julius-Maximilians-Universität Würzburg, GERMANY

## Abstract

Exam-related unwanted intrusive thoughts (UITs) and related neutralizing behaviors are common experiences among students. The present study investigated in what ways these UITs and behaviors are analogues to clinical obsessions and compulsions. Twenty-nine students completed three ecological momentary assessment surveys per day over 7 consecutive days, assessing the severity of exam-related UITs and related neutralizing behaviors, obsessive-compulsive (OC) symptoms, anxiety, distress, urge to neutralize, depressed mood, and stress in the week immediately before an exam period. Multilevel analysis demonstrated that the severity of exam-related UITs and related neutralizing behaviors was positively associated with OC symptoms, anxiety, distress, urge to neutralize, and stress but was not related to depressed mood. During the study period, the exam-related UITs occurred on average 7 times, and the related neutralizing behaviors on average 6 times. Overall, they were experienced with mild severity, low distress, and low urge to neutralize. Findings indicate that some aspects of exam-related UITs and related neutralizing behaviors (e.g., association with distress and urge to neutralize) might be analogous to OC symptoms but not all (e.g., no relation to depressed mood). We discuss how research on obsessive-compulsive disorder could benefit from considering exam-related UITs and related behaviors.

## Introduction

Almost everyone occasionally experiences thoughts that enter the mind abruptly and are perceived as unwanted [1, 2]. Such unwanted intrusive thoughts (UITs) occur on a variety of themes such as contamination fears, doubts about possible mistakes, harm, immoral sexual thoughts, religion, and symmetry or order [2–4]. Previous studies have demonstrated that UITs are ubiquitous and occur irrespective of cultural differences [2]. The cognitive model of obsessive-compulsive disorder (OCD) postulates that UITs lie on a continuum with obsessions [5, 6], since UITs share certain commonalities with clinically relevant obsessions [1]. Both UITs and obsessions are by definition repetitive intrusive thoughts, images, or impulses that are unwanted and disruptive [1, 7, 8]. Additionally, they tend to be followed by overt or covert neutralizing behaviors (termed compulsions in the context of OCD) with the aim of reducing the associated anxiety [7, 9, 10]. Previous studies suggest that the differences between UITs/neutralizing behaviors and obsessions/compulsions are a matter of degree rather than kind: The content is comparable [1, 3, 4, 11, however, see 12 for different findings], but UITs and neutralizing behaviors occur less frequently [1, 3, 11, 13] and are less distressing [1, 11, 13], and UITs interfere less with daily life than obsessions [3, 14].

It is common to study obsessive-compulsive (OC) phenomena in samples that do not necessarily include individuals with clinically relevant OC symptoms [15, 16]. This is mainly for practical reasons (easy to assess and affordable) but there are certain advantages as well [e.g., more precise experimental control; 15]. Two research practices are common. In the first, nonclinical participants are typically provided with a list of UITs and neutralizing behaviors and are asked to indicate which of these UITs and behaviors they have personally experienced [17–19]. One limitation of this method is that the participants tend to experience the selected UITs very rarely [e.g., a few times a year; 17, 20, 21], which might compromise the transfer of results to obsessions and compulsions. In the second practice, researchers temporarily induce UITs and neutralizing behaviors in the laboratory [16, 22]. For example, writing and then saying out loud a sentence such as “I hope [insert loved person] is in a car accident” can be an effective method of inducing UITs and neutralizing behaviors that resemble obsessions and compulsions [10, 16, 23, 24]. However, the experimentally induced UITs and neutralizing behaviors are not necessarily idiosyncratic (person specific) and do not occur naturally in everyday life. This might also limit the transfer of results to obsessions and compulsions, which are highly idiosyncratic [25] and typically occur spontaneously in various situations [21, 26]. Finding a way to study more frequently occurring, idiosyncratic UITs and related neutralizing behaviors in their natural settings would be a further benefit to analogue research on OCD, as it would address the limitations of the aforementioned two methods.

Exam taking in students might be a promising starting point for investigating naturally occurring idiosyncratic UITs and related neutralizing behaviors, since exam periods are characterized by several key factors that play a role in OCD: high levels of perceived anxiety, depression, and stress [27, 28]. During exam periods, students are faced with the threat of failing an important exam and it is their responsibility to prevent this threat from coming true; a critical mistake can have important consequences. The cognitive-behavioral conceptualization of OCD asserts that more anxiety and stress will lead to more frequent intrusions [5, 29]. This model also suggests that higher levels of depression will increase the probability of misinterpreting these intrusions, which in turn is likely to trigger neutralizing responses [29]. According to Salkovskis and Millar [30], perceived responsibility for the consequences of the intrusions is a prerequisite for a neutralizing behavior. Thus, it is plausible to assume that UITs and neutralizing behaviors that are analogues to obsessions and compulsions might occur frequently during exam periods. Previous studies found indications that university students experienced exam-related UITs during exam periods [31, 32], for example, thoughts related to inadvertently making critical mistakes [32], and that they performed related neutralizing behaviors, such as repetitively checking or superstitious behaviors [32, 33]. However, previous research did not address in what way these thoughts and behaviors might be analogous to obsessions and compulsions.

The main purpose of the present study was to evaluate in what ways naturally occurring, idiosyncratic exam-related UITs and related neutralizing behaviors can be considered analogues to clinical obsessions and compulsions. We consider mainly two aspects to be important for the evaluation: (a) the characteristics of the respective thoughts and behaviors, which are made explicit in their definitions, and (b) their associations with other theoretically relevant constructs. The strength of the associations would indicate the degree of overlap among these constructs. Thus, exam-related UITs and related neutralizing behaviors should share some typical characteristics with obsessions (e.g., intrusiveness) and compulsions (e.g., repetitiveness), respectively. In addition, they should be associated with OC symptoms and the core aspects of OCD [e.g., anxiety, distress, and urge to engage in neutralizing; 7, 29] to a medium to high degree and also with other relevant factors thought to play a role in OCD, such as depressed mood and stress [5, 29], to a somewhat lesser degree.

In this study, the key characteristics of exam-related UITs and related neutralizing behaviors were defined in a way that maximizes the chances of high conceptual overlap with clinically relevant obsessions and compulsions. We defined the idiosyncratic exam-related UITs as “intrusive, short thoughts, images, or impulses that pop into your mind repeatedly, are related to the exam and its preparations … [and are considered as] irrational, unwanted, or exaggerated” [32, p. 363]. Exam-related neutralizing behaviors were defined as any behaviors “dealing with such thoughts and/or the associated anxiety that was experienced as irrational or exaggerated” [32, p. 363]. Thus, by definition, exam-related UITs overlap with obsessive thoughts: Both are idiosyncratic, unwanted, intrusive, and irrational [7], enter the mind repeatedly, and can take the form of thoughts, images, or impulses. Similarly, exam-related neutralizing behaviors overlap with compulsions by definition: They tend to occur as a consequence of their respective unwanted intrusions as a way to manage the associated anxiety. In this study, we mainly focused on the second aspect to evaluate the analogy, that is, the associations of exam-related UITs and related neutralizing behaviors with OC symptoms and the core aspects of OCD, as well as other OCD-relevant factors.

To gain more insight into the experience of exam-related UITs and related neutralizing behaviors, we first collected descriptive information (type, frequency, degree of severity, distress, and urge to neutralize). Second, to evaluate the analogy, we investigated the associations of the exam-related UITs and related neutralizing behaviors with OC symptoms and the core aspects of OCD (anxiety, distress, and urge to neutralize) as well as other OCD-relevant factors (depressed mood and perceived stress). We hypothesized that the analogy would be reflected in medium to large positive associations of exam-related UITs and related neutralizing behaviors with OC symptoms, anxiety, distress, and urge to neutralize. In addition, we hypothesized that the exam-related UITs and related neutralizing behaviors would be positively associated with depressed mood and perceived stress but with lower than medium effect sizes, since depressed mood and stress are not symptoms of OCD but rather factors that might foster OCD. Additionally, we provide exploratory information about the potential adaptiveness of exam-related UITs and related neutralizing behaviors in terms of confidence in passing the exam, and about the context in which the exam-related UITs and related neutralizing behaviors typically occur.

For brevity, in the Methods and Results sections, we use the abbreviations ER for exam related and N for neutralizing behaviors, for example, ER-UITs-N when referring to exam-related UITs and related neutralizing behaviors as one construct and ER-UITs and ER-N for exam-rated UITs and exam-rated neutralizing behaviors, respectively.

## Methods

### Participants and measures

We recruited 29 undergraduate psychology students (*M*_age_ = 21.36 years, *SD* = 1.87; 92.86% female) over 3 consecutive years at the end of the participants’ 1st year at the University of Basel, 1 week before a stressful exam period. Participants were a subsample of the Wahl, Hofer (32) study who agreed to participate in the ecological momentary assessment (EMA) part. The study was approved by the Ethics Committee of the Department of Psychology, University of Basel. Participants received course credit and monetary compensation (20 Swiss francs) for their participation.

#### Baseline measures

*Depression and anxiety (baseline)*. We used the Beck Depression Inventory [BDI; 34, German version: 35] and the Beck Anxiety Inventory [BAI; 36, German version: 37] to assess depressive and anxiety symptoms, respectively. Each scale consists of 21 items. The psychometric properties of the scales are well established [36–38].

*OC symptoms (baseline)*. The Obsessive-Compulsive Inventory, Revised [OCI-R; 39, German version: 40], an 18-item self-report measure, was used to assess OC symptoms. The scale has demonstrated good validity and reliability [40, 41].

*Perceived stress (baseline)*. The Symptoms subscale (13 items) of the Stress and Coping Inventory [SCI; 42] was used to assess perceived stress at baseline. SCI is a self-report questionnaire regarding perceived stress, stress symptoms, and coping with stress. The SCI Symptoms subscale measures physiological (e.g., abdominal pain, headache) and psychological (e.g., concentration and sleep problems) stress symptoms in the last 6 months. The subscale has good validity and reliability [32, 42].

*Types of most frequent ER-UITs and ER-N (baseline)*. We identified the two most frequent ER-UITs and the two most frequent ER-N for each participant using the Stress-Related Thoughts and Behavior List [StressRTBL; 32]. Using this list, the interviewer read the definition of ER-UITs-N and then administered a checklist of 47 items assessing the occurrence of ER-UITs and ER-N over the past 2 weeks. The ER-UITs and ER-N were categorized into three and five groups of similar themes, respectively. The thought categories were fear of forgetting something important (e.g., accidently missing a whole book chapter), fear of making a critical mistake (e.g., accidently mixing up documents), and superstitious thoughts (e.g., must study for a specific amount of time per day or the exam will not go well). The behavior categories were checking (e.g., checking exam material to make sure nothing important was left out), ordering (e.g., arranging the desk), superstitious behavior (e.g., always using the same pen while studying), rigid rules or rituals (e.g., organizing every day in detail), and reassurance seeking (e.g., seeking reassurance that the exams will go well). Participants were asked to choose from the checklist the two ER-UITs and two ER-N that they had experienced most frequently in the last 2 weeks. These most frequent UITs and behaviors were then used for the EMA.

#### EMA measures (administered with a self-developed EMA app on a smartphone)

*Severity of ER-UITs-N (EMA)*. We administered a modified Yale–Brown Obsessive Compulsive Scale [Y-BOCS; 43, German version: 44] to assess the severity of ER-UITs (modified Y-BOCS Obsessions subscale; items 1–5) and ER-N (modified Y-BOCS Compulsions subscale; items 6–10) in the last 30 min. Participants were asked to think about their first or second most frequent ER-UIT or ER-N, as identified in the StressRTBL interview, when answering. This procedure was preferred over asking about “any ER-UIT/ER-N” to provide a clear reference and thus increase the validity of the method. The answers were recorded on a scale of 0 (*not at all severe*) to 4 (*extremely severe*). It is common to investigate obsessions and compulsions as one construct [45], since they cooccur [46]. Thus, we operationalized the EMA severity of ER-UITs-N as the total scale of the modified Y-BOCS (sum score of items 1–10).

*Frequencies of ER-UITs and ER-N (EMA)*. We assessed frequency by referring to the two most frequent ER-UITs and ER-N in the last 30 min, as identified during the baseline StressRTBL interview (“How often did you experience thoughts such as [most frequent ER-UIT] or [second most frequent ER-UIT] in the last 30 minutes?” “How often did you engage in behaviors such as [most frequent ER-N] or [second most frequent ER-N] in the last 30 minutes?”). Both items were rated on a scale of 0 (not at all) to 5 (always).

*Perceived anxiety*, *depressed mood*, *distress*, *and urge to neutralize (EMA)*. We assessed anxiety (“How afraid were you in the last 30 minutes?”) and depressed mood (“To what degree did you feel depressed or despondent in the last 30 minutes?”) with one item each on a scale of 0 (*not at all*) to 100 (*extremely*). Distress (“To what degree did you feel distressed in response to your [first most frequent ER-UIT] or [second most frequent ER-UIT] in the last 30 minutes?”) and urge to neutralize (“To what degree did you experience the urge to give in to your intrusive exam-related thoughts or to reassure yourself with an action such as [first most frequent ER-N] or [second most frequent ER-N] in the last 30 minutes?”) were both rated on a scale of 0 (*very low*) to 5 (*extremely high*).

*OC symptoms (EMA)*. We used the modified OCI-R to assess OC symptoms in the last 30 min. In contrast to the baseline measure, we did not include the Hoarding subscale. Included subscales were washing, obsessing, ordering, checking, and neutralizing (mental counting rituals). The answer categories ranged from 0 (*not at all*) to 5 (*all the time*).

*Perceived stress (EMA)*. We assessed overall level of stress (“How stressed were you in the last 30 min?”) with one item, rated on a scale of 0 (*not at all*) to 5 (*extremely*).

*Perceived likelihood of exam success*, *studying for exams*, *and presence of other people (EMA)*. To assess the perceived likelihood of exam success, the following question was administered: “How likely is it that you will pass the upcoming exams?” The answers were provided on a scale of 0% to 100%. Studying for exams (“Are you currently reviewing materials for the upcoming exams?”) and the presence of other people (“Are you currently alone?”) were both answered with either 1 (*yes*) or 0 (*no*).

### Procedure

The study took place during the week before a critical 2-week exam period at the end of the participants’ 1st year at the University of Basel. If students fail any of the six exams more than once, they are not allowed to study psychology anywhere in Switzerland. Therefore, this time period represents a very stressful life event for the students. Our testing started with a baseline measure and was followed by a 1-week EMA. For the baseline assessment, participants were tested individually in a laboratory at the University of Basel. All participants gave their written informed consent to participate in the study. Participants were then asked to rate their anxiety, depressed mood, OC symptoms, and perceived stress on a set of standardized questionnaires (BAI, BDI, OCI-R, and SCI). Following this, they were administered the StressRTBL by trained master’s students to identify their two most frequent ER-UITs and two most frequent ER-N (these were used as the target thoughts and behaviors for the EMA survey). After completing the baseline assessment, they were introduced to the EMA app, which was installed on a smartphone. Subsequently, they completed a short practice trial to ensure they understood how to respond correctly to the EMA surveys. The investigator created an EMA schedule for every participant. During the next 7 days (the EMA period), the participants were prompted three times a day (Time Point 1: 7–10 a.m., Time Point 2: noon–5 p.m., and Time Point 3: 4–11 p.m.) to self-evaluate the frequency, distress, urge to neutralize, and severity of their two most frequent ER-UITs and two most frequent ER-N as well as anxiety, depressed mood, OC symptoms, perceived stress, and additional variables (perceived likelihood of exam success, studying for exams, and presence of other people). For two participants, the scheduled EMA surveys for the morning hours conflicted with their private schedule. Thus, they were prompted in the early afternoon (noon–1 p.m.), late afternoon (3–5 p.m.), and evening (6–10 p.m.).

### Statistical analysis

All data were analyzed with IBM SPSS Statistics version 25; the figure was created using GraphPad Prism version 8.2.0 for Windows. First, we provide descriptive information about the type, frequency, and severity of ER-UITs-N as well as distress and urge to neutralize. To determine the frequency of ER-UITs during the EMA period, we transformed the frequency measure of these UITs into a dummy variable with ratings of 0 (*not at all*) coded as 0 (*ER-UIT did not occur*) and ratings of 1–5 coded as 1 (*ER-UIT occurred*). Subsequently, we built a sum score across all 21 time points for each participant. The frequency of ER-N during the EMA period was operationalized the same way as the frequency of ER-UITs.

Second, we conducted multilevel analyses to examine the associations of ER-UITs-N with the OC symptoms, OCD-relevant factors, and additional variables (perceived likelihood of exam success, studying for exams, and presence of other people). Specifically, we calculated separate multilevel models for the EMA severity of ER-UITs-N (modified Y-BOCS total scale) as the outcome variable with each of the nine predictors. Predictors were EMA OC symptoms, anxiety, distress, urge to neutralize, depressed mood, perceived stress, perceived likelihood of exam success, studying for exams, and presence of other people.

Consistent with current recommendations [47, 48], we kept the random effects at the justifiable maximum. Thus, for each separate model, we defined the intercept and slope as random effects. For each multilevel analysis, we constructed a two-level model with the measurement occasions (*n* = 21 per participant; Level 1) nested within individuals (Level 2). Each model contained the respective predictor plus the variable time (in days) to account for linear time trends. As both of these variables were time varying, we added a random slope parameter for both of them. However, if the model failed to converge, we removed the random slope of the main predictor. For brevity, we do not report the time variable in the Results section.

To estimate all parameters, we used the maximum likelihood estimation method. Unstandardized estimates are reported in the Results section. Consistent with recent recommendations [49, 50], we calculated Cohen’s *f*^2^ [51] as the indicator of effect size for the fixed effects. Cohen’s *f*^2^ can be interpreted as the proportion of variance accounted for by the given predictor relative to a null model. We report the *f*^2^ value unique to each main predictor, that is, over and above the effect of the variable time on the outcome variable. To ensure that the reduction of variance was accounted for only by the fixed effects and not by the random effects, we held the random effects constant (i.e., we defined only a random intercept) for each model [50] when calculating *f*^2^. The magnitude of *f*^2^ can be interpreted using Cohen’s guidelines [51]: 0.02 for a small effect, 0.15 for a medium effect, and 0.35 for a large effect; the in-between *f*^2^ values were interpreted as a range (e.g., a value of 0.08 would be interpreted as a small to medium effect; values above 0.35 were interpreted as large effects). The continuous EMA predictors were group-mean centered to facilitate interpretation, such that the obtained effects represented changes in the outcome due to changes on the individualized predictor scale, that is, both slope and intercept. Visual inspection of the data did not indicate a violation of the models’ assumptions (functional form, normality of residuals, and homoscedasticity). The level of statistical significance was set at *p* < .05.

Following the generalizability approach [52] and consistent with current recommendations [53], we computed between- (*R*_*KF*_) and within-person (*R*_*C*_) reliability coefficients for the EMA scales (modified Y-BOCS and modified OCI-R). While *R*_*KF*_ represents the reliability of a measure across all days, *R*_*C*_ is the reliability of change in ratings over time across individuals. The reliability coefficients from .61 to .80 were interpreted as moderate and from .81 to 1.00 as substantial [54].

## Results

### Sample characteristics, compliance with the EMA method, and reliability coefficients

Two participants were excluded from the main analyses because they reported intrusive thoughts that were more typical of worrisome thoughts [55; e.g., “What will happen if I don’t pass the exam?” or “What is my plan B?”] instead of ER-UITs during the baseline interview (StressRTBL). Table 1 shows means, standard deviations, and reliability coefficients for baseline measures of OC symptoms, anxiety, depression, and perceived stress. The OC symptoms appeared comparable to those reported in other studies with university students [56–58, however, see 59, 60 for different findings]. Anxiety and depressive symptoms in this sample seemed comparable to those in another student sample under exam stress [61]. The internal consistency of the baseline measures ranged from good (SCI symptoms) to excellent (BAI). The participants completed a total of 92.06% of the possible EMA prompts, which suggests a high compliance with the study design [for comparison, see 62, 63]. The reliability coefficients for both EMA scales were substantial (modified Y-BOCS: *R*_*KF*_ = 1.00, *R*_*C*_ = .90; modified OCI-R: *R*_*KF*_ = .99, *R*_*C*_ = .82).

**Table 1:** Means, standard deviations, and Cronbach’s alpha of the baseline measures.

Baseline variable	*M* (*SD*)	α
OC symptoms (OCI-R)	11.04 (9.54)	.89
Anxiety (BAI)	14.15 (9.29)	.90
Depression (BDI)	8.04 (6.88)	.89
Perceived stress (SCI symptoms)	24.81 (6.82)	.84

*Note*. BAI = Beck Anxiety Inventory; BDI = Beck Depression Inventory; OC = obsessive-compulsive; OCI-R = Obsessive-Compulsive Inventory, Revised; SCI = Stress and Coping Inventory.

### Descriptive information about the ER-UITs and ER-N (type, frequency, degree of severity, distress, and urge to neutralize)

**Fig 1:**
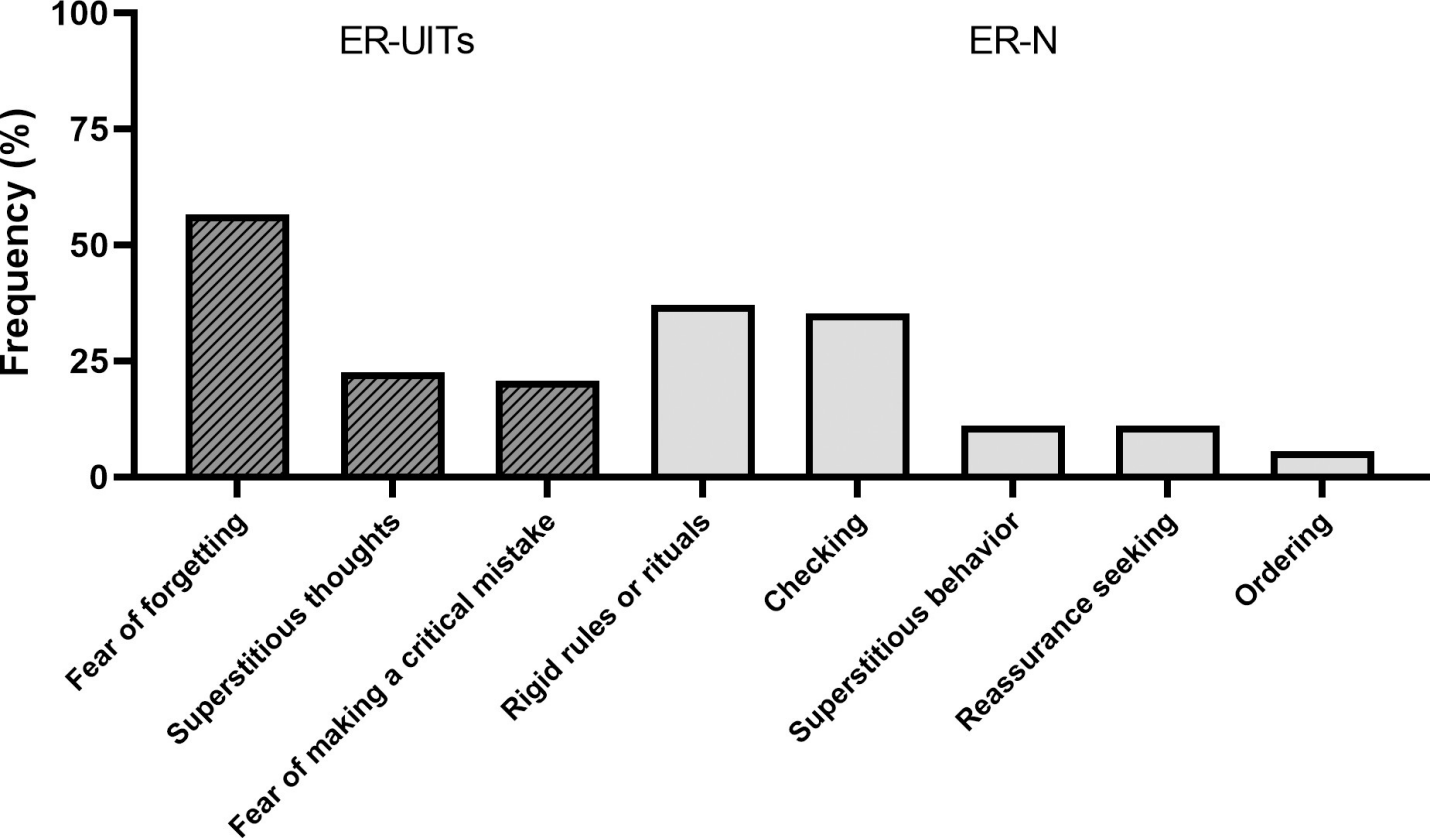
Frequencies (as percentages) of the most frequent exam-related unwanted intrusive thoughts (ER-UITs) and exam-related neutralizing behaviors (ER-N) as reported by the participants on the stress-related thoughts and behavior list at baseline. The frequency represents the number of each reported UIT and behavior within the total number reported (for ER-UITs: *n* = 53; for ER-N: *n* = 54) by the participants. Each participant could report a maximum of two UITs as well as two behaviors.

Fig 1 shows the most frequent types of ER-UITs and the most frequent types of ER-N as indicated by the participants during the baseline StressRTBL interview. Each participant could report a maximum of two ER-UITs and two ER-N. In total, 53 ER-UITs and 54 ER-N were reported. The most prominent ER-UITs were the fear of forgetting something important, followed by superstitious thoughts and the fear of making a critical mistake. The most prominent ER-N were rigid rules or rituals, followed by checking, superstitious behavior, reassurance seeking, and ordering. During the EMA period, on average (across all subjects and time points), participants experienced seven ER-UITs (*M* = 6.67, *SD* = 5.06) and six ER-N (*M* = 5.96, *SD* = 4.85).

Table 2 shows means and standard deviations of the severity of ER-UITs-N, distress, and urge to neutralize, and the number of occurrences of low, mild, moderate, and high scores of the severity of ER-UITs-N during the EMA period. On average, participants experienced the ER-UITs-N with mild severity, low distress, and low urge to neutralize.

**Table 2:** Means and standard deviations of severity of ER-UITs-N, distress, and urge to neutralize, and number of occurrences of low, mild, moderate, and high scores of the severity of ER-UITs-N during the EMA period.

EMA variable	*M* (*SD*)^a^	*N* (%)^b^
Severity of ER-UITs-N^c^	8.79 (6.37)	
Distress	1.49 (1.08)	
Urge to neutralize	1.31 (1.04)	
Severity score of ER-UITs-N		
Low (scores of 0–7)		52 (44.07)
Mild (scores of 8–15)		50 (42.37)
Moderate (scores of 16–23)		13 (11.02)
High (scores of 24–40)		3 (2.54)

*Note*. EMA = Ecological momentary assessment; ER-UITs-N = exam-related unwanted intrusive thoughts and related neutralizing behaviors. Y-BOCS = Yale–Brown Obsessive Compulsive Scale.

^a^ To calculate the means, we included only those EMA prompts in which the participants reported experiencing the ER-UIT together with the ER-N in the last 30 min.

^b^ To calculate the number of occurrences of low, mild, moderate, and high scores of the severity of ER-UITs-N, we included only those EMA prompts in which the participants reported experiencing the ER-UIT together with the ER-N in the last 30 min. The benchmarks for the severity were consistent with the common Y-BOCS cut-offs [45].

^c^ Scoring is consistent with Y-BOCS, e.g., 8–15 = mild symptom severity [45].

### The associations of ER-UITs-N with OC symptoms, anxiety, distress, urge to neutralize, depressed mood, perceived stress, perceived likelihood of exam success, studying for exams, and presence of other people (EMA measures)

Table 3 displays the results of the multilevel analyses. The severity of ER-UITs-N was positively associated with OC symptoms, anxiety, distress, and urge to neutralize, respectively. The associations with OC symptoms and anxiety were small to medium in size, while the associations with distress and urge to neutralize were large. The severity of ER-UITs-N was positively associated with perceived stress but was not related to depressed mood. The degree of association with perceived stress was small to medium.

**Table 3:** Associations between the EMA severity of ER-UITs-N (outcome) and other EMA variables (predictors).

EMA predictor variable (fixed effects)	Severity ER-UITs-N (modified Y-BOCS total)		
	Coefficient (*SE*)	*p*	*f* ^2^
OC symptoms	0.46 (0.13)	.004	.06
Anxiety	0.07 (0.02)	.002	.14
Distress	2.73 (0.34)	< .001	.58
Urge to neutralize	2.68 (0.34)	< .001	.50
Depressed mood	0.04 (0.02)	.052	.03
Perceived stress	1.17 (0.37)	.004	.12

*Note*. Each fixed effect represents a separate multilevel analysis with EMA severity of ER-UITs-N as the outcome. EMA = Ecological momentary assessment; ER-UITs-N = exam-related unwanted intrusive thoughts and related neutralizing behaviors; OC = obsessive-compulsive; Y-BOCS = Yale–Brown Obsessive Compulsive Scale.

The severity of ER-UITs-N was negatively associated with the perceived likelihood of exam success (coefficient = -0.07, *SE* = 0.02, *p* < .001, *f*^2^ = .03) and positively associated with studying for exams (coefficient = 1.15, *SE* = 0.38, *p* = .005, *f*^2^ = .02). There was no association between the severity of ER-UITs-N and presence of other people (coefficient = -0.04, *SE* = 0.47, *p* = .940, *f*^2^ < .001).

## Discussion

To present a more comprehensive picture of the experience of exam-related UITs and related neutralizing behaviors, we first provide descriptive information about this phenomenon. The most common type of exam-related UITs reported by the students was the fear of forgetting something important, followed by superstitious thoughts and the fear of making a critical mistake. The most common exam-related neutralizing behaviors was rigid rules or rituals closely followed by checking, then superstitious behaviors and reassurance seeking, and finally ordering. During the study period, students reported experiencing the exam-related UITs on average seven times and engaging in related neutralizing behaviors on average six times. Compared to OC symptoms, which occurred several times a day in other studies [1, 13], exam-related UITs and related neutralizing behaviors seem to be less frequent. However, when we compare them roughly to the occurrence of subclinical OC symptoms, which were experienced on average once or twice a month in Morillo, Giménez [64], exam-related UITs and related neutralizing behaviors seem to be more frequent than subclinical OC symptoms.

The exam-related UITs and related neutralizing behaviors were experienced on average with mild severity, low distress, and low urge to neutralize. Since the scoring of the severity of these UITs and related behaviors is consistent with the Y-BOCS [45], exam-related UITs and related neutralizing behaviors might be comparable to mild OC symptom severity. In other studies, OC symptoms were on average associated with moderate to high distress and moderate to high urge to neutralize [1, 65]. In light of this, exam-related UITs and related neutralizing behaviors appear to be less distressing, and the urge to neutralize is lower than for OC symptoms. We emphasize that the previous studies used different methods to assess the frequency, severity, distress, and urge to neutralize related to OC symptoms, and thus our comparisons should be considered only rough estimations. Future studies are needed to directly compare exam-related UITs and related neutralizing behaviors with OC symptoms on frequency, severity, distress, and urge to neutralize.

The main goal of the present study was to investigate in what ways idiosyncratic exam-related UITs and related neutralizing behaviors can be considered analogues to clinical obsessions and compulsions. To evaluate the analogy, we primarily focused on the associations of the exam-related UITs and related neutralizing behaviors with OC symptoms and the core aspects of OCD, as well as with other OCD-relevant factors. The positive associations of the severity of exam-related UITs and related neutralizing behaviors with OC symptoms, anxiety, distress, urge to neutralize, and perceived stress generally support a conceptual overlap of these UITs and behaviors with obsessions and compulsions. As predicted, the associations with distress and urge to neutralize were medium to large in their effect sizes. However, the associations with OC symptoms were smaller than expected. A possible explanation might be that common types of exam-related UITs and related neutralizing behaviors reported in this study did not exactly match the symptom dimensions included in the OCI-R (the scale we used to assess OC symptoms). For example, the OCI-R does not assess superstitious thoughts that were prominent exam-related UITs and are also common in OCD [4, 66]. Future studies might want to investigate the associations of exam-related UITs and related neutralizing behaviors also with other measures of OC symptoms, such as the Obsessional Intrusive Thoughts Inventory [4], which also includes a superstition dimension. The relatively small association of the severity of exam-related UITs and related neutralizing behaviors with anxiety was also not consistent with our hypothesis. Previous studies have demonstrated medium to large associations between OC symptoms and anxiety [40, 43]. The severity of exam-related UITs and related neutralizing behaviors was not associated with depressed mood. Other studies have shown OC symptoms to be related to depressed mood [67–69]. Overall, our hypotheses were partially supported.

Additionally, we investigated factors that might be associated with the exam-related UITs and related neutralizing behaviors. An increase in the severity of these UITs and behaviors was associated with a decrease in the perceived likelihood of passing the upcoming exams. This indicates that exam-related UITs and related neutralizing behaviors might not be adaptive for students in terms of passing the exams. Future studies might want to investigate the effect of these UITs and behaviors on *actual* exam outcomes. Students who were currently studying for the upcoming exams experienced more severe exam-related UITs and related neutralizing behaviors than those who were not currently studying. This indicates that the occurrence of exam-related UITs and related neutralizing behaviors might not be evenly distributed during exam periods but might be more frequent when actually studying. However, this finding must be interpreted with caution, since we did not measure the duration of exam preparations. Finally, the severity of exam-related UITs and related neutralizing behaviors did not change depending on the presence of other people.

Whether exam-related UITs and related neutralizing last beyond the exam period is an interesting question that is also relevant for future research. Since previous studies showed that exam-related UITs decreased once the exam was completed [31] and that magical rituals occurred with much higher frequency directly before an exam compared to a regular study day [33], it is plausible to assume that they would typically not last beyond the exam period. However, factors such as dysfunctional interpretations of exam-related UITs or reductions of distress after neutralizing, resulting in negative reinforcement mechanisms, might contribute to their persistence. These factors are interesting targets of further longitudinal research.

There are several limitations of the current study to acknowledge. First, although we investigated important associations of exam-related UITs and related neutralizing behaviors with OC symptoms and the core aspects of OCD, we did not directly address the degree of overlap in some of the defining characteristics, such as the degree of intrusiveness, irrationality, or ego-dystonicity [21]. Second, the findings might not generalize beyond our sample to the population. Next, we did not control for reactivity, that is, participants’ biased responses solely due to the EMA methodology. However, recent studies indicated that the effect of smartphone assessment on the frequency of intrusions is rather low [62, 70]. Finally, the psychometric properties of the single-item measures we used to assess a number of relevant constructs (e.g., anxiety, depression, etc.) during the study period should be explored.

To conclude, exam-related UITs and related neutralizing behaviors might be considered analogous to obsessions and compulsions in the sense that they are accompanied by distress and an urge to neutralize and might be susceptible to stress. However, they might not be considered analogues to obsessions and compulsions in the sense that their relation to anxiety is only small, and they are also not related to depressed mood. The conceptual overlap with obsessions and compulsions was smaller than expected, which might be accounted for by the assessment methods.

We suggest that exam-related UITs and related neutralizing behaviors could extend analogue research by providing an opportunity to study relatively frequent, idiosyncratic UITs and related neutralizing behaviors in their natural environment. Relative to questionnaire studies, which typically use UITs with a very low frequency, exam-related UITs have the advantage of being more frequent, and their variation over time could be used to study covariations with potential factors that might foster OCD and their mutual or converging courses in longitudinal studies. For example, a longitudinal study design could be used to investigate to what extent stress is involved in the occurrence and recurrence of UITs, possibly as a potentiator of dysfunctional interpretations. This might help further elucidate how obsessions and compulsions develop in the first place. Additionally, experimental studies could investigate the fine-grained interactions between the occurrence of intrusive thoughts or images, negative emotional reactions, and repetitive responses to reduce these thoughts and emotions during episodes of exam-related UITs and neutralizing behaviors. This might eventually be helpful in clarifying how some compulsive episodes become excessively long. Exam-related UITs and neutralizing behaviors might be less useful in studies that investigate questions related to anxiety or depression in OCD, since their associations might not reflect the relation of OC symptoms with these constructs. Additionally, the application of exam-related UITs might be less relevant for laboratory-based experimental studies, since our preliminary data suggest that they occur more often when studying for exams.
